# Reshaping the social meaning of smoking: the UK’s tobacco-free generation policy

**DOI:** 10.1093/eurpub/ckag107

**Published:** 2026-07-12

**Authors:** Britta K Matthes, Jon Berrick

**Affiliations:** Department for Health, University of Bath, Bath, United Kingdom; SMRI, The University of Sydney, Sydney, NSW, Australia

The UK’s tobacco-free generation policy does more than simply restrict future tobacco sales. It should also be seen as a policy intended to reshape how smoking is socially understood. Under the Tobacco and Vapes Act [1], people born on or after 1 January 2009 will never be legally sold or supplied tobacco. Unlike fixed age limits, which imply that smoking becomes acceptable in adulthood, this approach removes the idea that there is any stage of life at which smoking is acceptable. That shift reflects a move from controlling smoking uptake towards policies that seek to progressively denormalize smoking, through what has been described as a ‘generational firebreak’ [2].

Despite substantial progress in tobacco control, tobacco use still causes around 80 000 deaths annually in the UK [3] and 500 000 in the EU [4], and most people who smoke start in adolescence [2]. For decades, prevention efforts have focused on discouraging uptake among young people. Traditional minimum age laws have been central to this approach. However, they also position smoking as an adult behaviour–something one grows into.

Internal tobacco industry documents indicate that tobacco companies have understood this dynamic well. In the context of widespread age restrictions, industry-commissioned research described cigarettes as ‘the entrance ticket to the hall of the adult society’ [5], reinforcing their appeal to young people seeking to signal personal maturity. Unsurprisingly, companies have historically argued for conventional minimum age laws, particularly those with lower age thresholds [3], while also using them to present themselves as acting responsibly on youth smoking and to resist further regulation.

The tobacco-free generation approach [6]—also called a generational sales ban—takes a different path. Rather than establishing a threshold age at which smoking becomes acceptable, it sends a clear message that because there is no age at which smoking is safe, there is none at which it should be considered acceptable [7].

Over time, this could have important effects because public health laws may be most effective when legal restrictions align with—and positively influence—broader social and behavioural norms. As the pre-2009 cohorts age, their smoking is likely to become progressively less salient as a behavioural reference point for new adolescent cohorts. Moreover, smoking may gradually become less visible. Importantly, the approach does not impose restrictions on those already addicted, for whom the UK government has instead been increasing funding for cessation support [3].

The ‘no safe age’ message communicated by the tobacco-free generation policy may help explain why the approach has attracted strong public support, including among a majority of people who smoke [8]. It also builds on decades of tobacco control measures in the UK—from smoke-free public places to standardized packaging–that have progressively reduced the social acceptability of smoking.

These normative implications of the approach can also help explain the response from the tobacco industry, widely recognized as a major barrier to tobacco control. Analysis of submissions from tobacco companies and linked actors to the UK public consultation and call for evidence shows clear opposition to the generational sales ban [9]. Instead, these actors advocated for alternatives such as raising the age of sale to 21 or exempting certain products. As with previous tobacco control measures, familiar arguments have reappeared: warnings about illicit trade, concerns for retailers, and allegations that the evidence base is insufficient. Previous experience in Balanga, Philippines, and Brookline, USA, suggests that such policies may attract legal challenges from the tobacco industry [10]. Taken together, this opposition to measures that would ultimately end tobacco sales contradicts the industry’s broader ‘transformation’ narrative of supporting a transition away from combustible tobacco.

With the passage of the UK Act, attention has turned to implementation. Since the tobacco-free generation policy focuses on sale and supply, effective enforcement, including through proposed retail licensing measures, is likely to be important. Continued investment in smoking cessation will also remain crucial. Effective communication will matter too. If the policy is framed merely as another youth restriction rather than as a broader norm-shaping public health measure, its normative impact may be diluted. If, instead, it is understood as a statement that tobacco is not a product future generations should ever need, it has greater potential to reinforce broader social norms against tobacco use.

The UK is the third country to adopt a tobacco-free generation policy aimed not just at reducing or delaying smoking uptake, but at progressively making smoking obsolete for future generations. Similar provisions were previously enacted in New Zealand (controversially repealed after a change of government) and, more recently, the Maldives [10]. Momentum has also been building at local level, particularly in the US, where >20 localities have introduced similar policies since 2021 [10].

The UK’s experience will therefore be closely observed internationally, especially in Europe, where Europe’s Beating Cancer Plan aims to ensure that <5% of the population uses tobacco by 2040 [10]. The policy has already attracted attention in several European countries, including France and Spain, alongside wider momentum around tobacco endgame strategies across Europe [10].

The success of the policy will depend not only on effective enforcement, but also on the extent to which it reshapes how smoking is understood. If successful, the UK approach could provide an important model for European endgame policies that seek to shift expectations around smoking, rather than simply restrict youth access to tobacco.

Conflict of interest: The authors declare no conflicts of interest.

## Data Availability

All sources on which this viewpoint is based are publicly available.
